# Nuclear Export of Cyclin B Mediated by the Nup62 Complex Is Required for Meiotic Initiation in *Drosophila* Males

**DOI:** 10.3390/cells9020270

**Published:** 2020-01-22

**Authors:** Ryotaro Okazaki, Kanta Yamazoe, Yoshihiro H. Inoue

**Affiliations:** Department of Insect Biomedical Research, Center for Advanced Insect Research Promotion, Kyoto Institute of Technology, Kyoto, Japan; chiri.tsumo.99@gmail.com (R.O.); kantavery.mj.1227@gmail.com (K.Y.)

**Keywords:** cell cycle, male meiosis, Nup62, cyclin B, *Drosophila*

## Abstract

Background: The central channel of the nuclear pore complex plays an important role in the selective transport of proteins between the nucleus and cytoplasm. Previous studies have demonstrated that the depletion of the Nup62 complex, constructing the nuclear pore channel in premeiotic *Drosophila* cells, resulted in the absence of meiotic cells. We attempted to understand the mechanism underlying the cell cycle arrest before meiosis. Methods: We induced dsRNAs against the nucleoporin mRNAs using the Gal4/UAS system in *Drosophila*. Results: The cell cycle of the *Nup62*-depleted cells was arrested before meiosis without CDK1 activation. The ectopic over-expression of CycB, but not constitutively active CDK1, resulted in partial rescue from the arrest. CycB continued to exist in the nuclei of *Nup62*-depleted cells and cells depleted of exportin encoded by *emb*. Protein complexes containing CycB, Emb, and Nup62 were observed in premeiotic spermatocytes. CycB, which had temporally entered the nucleus, was associated with Emb, and the complex was transported back to the cytoplasm through the central channel, interacting with the Nup62 complex. Conclusion: We proposed that CycB is exported with Emb through the channel interacting with the Nup62 complex before the onset of meiosis. The nuclear export ensures the modification and formation of sufficient CycB-CDK1 in the cytoplasm.

## 1. Introduction

Approximately 1000 nuclear pore complexes (NPCs) are present in the nuclear envelope of eukaryotic cells. These small pores, which span the double membrane nuclear envelope, play an important role in the regulation of the nuclear-cytoplasmic transport of mRNAs and proteins [1,2,3]. The proteins constructing the NPC are known as nucleoporins (Nups). More than 30 types of Nups have been identified, and these are highly conserved among eukaryotes, from yeast to human [4,5]. The NPC is roughly divided into three domains, such as the cytoplasmic filament, nuclear basket, and central core [6,7]. The cytoplasmic filament is a fibrous protrusion extending toward the cytoplasm and consists of the Nup214 complex and Nup358. The nuclear basket, which projects toward the nucleoplasm, consists of Tpr, Nup153, and Nup50. The central core is a cylindrical structure embedded in the nuclear envelope. This sub-domain is subdivided into a skeletal part and a central channel constructing the lumen of the pore. The skeletal part is constructed by the Y-complex containing the Nup107-106 and Nup93 complexes, and this structure is fastened to the nuclear envelope with several Nups, such as Ndc1, Gp210, and Pom121. In addition, the central channel is constructed by the Nup62 complex, which consists of three Nups, namely, Nup62, Nup58, and Nup54 in *Drosophila*. These Nups, designated as FG-Nups, contain the phenylalanine-glycine (FG) repeat domain [8,9]. FG-Nups bind to proteins that shuttle between the nucleus and cytoplasm [10,11]. The FG-barrier constructed by the FG-Nups allows the selective transport of these proteins [12,13,14].

As most of the Nups are well-conserved in *Drosophila*, we decided to investigate the role of the NPC in spermatogenesis using this model animal that allows well-advanced genetic analyses [15,16,17]. A previous study using spermatocyte-specific RNAi experiments investigated whether the depletion of the 30 Nups affected the progression of premeiotic cells through meiosis [17]. The authors reported that the depletion of all three members of the Nup62 complex, but not of other Nups, resulted in the absence of meiotic cysts in their testes. However, the mechanism underlying the absence of meiotic cells in testes depleted of the Nup62 complex is not known. *Drosophila* spermatogenesis commences from the unequal division of germline stem cells localized at the tip of the testis. After four rounds of mitosis of the spermatogonium, one of the two daughter cells of the stem cells differentiate, and the resultant 16 cells form a single cell unit called the cyst. Every spermatocyte in a cyst undergoes cell growth synchronously, and subsequently they initiate the first meiotic division at the same time. Consequently, 32 cells are generated after the completion of the first meiotic division. Finally, 64 post-meiotic cells called spermatids are made in the cyst [16,18,19,20]. Whether meiotic divisions have been performed twice, once, or not even once can be judged by counting the number of post-meiotic cells present in a spermatid cyst.

CDK1 plays the most important role in the initiation of both mitotic and meiotic cell division. In eukaryotes, the following three conditions are essential for the activation of the protein kinase that triggers cell division [21]: (1) complex formation of CDK1 with its regulatory subunit, cyclin B (CycB), (2) phosphorylation of the threonine residue at the 161th amino acid from the N-terminal (Thr^161^) of CDK1, and (3) removal of phosphate groups from the 14-threonine (Thr^14^) and 15-tyrosine (Tyr^15^) of CDK1, both of which are involved in negative regulation of the kinase [22,23,24,25]. Thr^161^ of CDK1 is phosphorylated by the CDK-activating kinase (CAK) in the nucleus [26]. Subsequently, the complex is exported from the nucleus and it accumulates in the cytoplasm. The phosphate groups at Thr^14^ and Tyr^15^ of CDK1 are removed in the nucleus by a Cdc25 orthologue encoded by *twe* and the CDK1 is activated in the premeiotic cells. To modify the CycB and ensure a sufficient amount of the complex, the protein is exported to the cytoplasm. The nuclear export signal (NES) in the cytoplasmic retention signal (CRS) of CycB plays a critical role in nuclear export. CRM1, one of exportins, recognizes the NES and exports the CycB to the cytoplasm via interaction with the sequences [27,28,29]. 

In this study, we confirmed that a spermatocyte-specific depletion of all the members of the Nup62 complex, namely, Nup54, Nup58, and Nup62, resulted in the cell cycle arrest of premeiotic cells before the first meiotic division. Immunostaining demonstrated that the failure of meiotic entry resulted from the inhibition of CDK1 activation in cells prior to meiosis. As genetic evidence indicated that phosphorylation and dephosphorylation of CDK1 were not involved in cell cycle arrest, we investigated the cellular localization of CycB. Consequently, we observed that the regulatory subunit for CDK1 remained to be accumulated in the nuclei of the premeiotic cells. These observations suggested that absence of active CDK1 in *Nup62*-depleted cells resulted from abnormal localization of the complex, which is responsible for the failed nuclear export of the protein. We showed that a *Drosophila* CRM1 orthologue encoded by *emb* is required for CycB export. We further observed temporal protein complexes containing CycB, Emb, and Nup62 in the premeiotic cells. Overall, we proposed that selective export of CycB through the NPC is required to initiate male meiosis in *Drosophila* spermatogenesis. 

## 2. Materials and Methods

### 2.1. Drosophila Stocks

For a depletion of three members consisting of Nup62 complex in NPC, we used the following UAS-RNAi stocks. To induce expression of dsRNA for *Nup62*, *P{TRiP.GLV21060}attP2* (#35695), *P{TRiP.GL01533}attP40* (#43189) from Bloomington Drosophila Stock Center (BDSC) (Bloomington, IN, USA) and *P{KK108318}VIE-260B* (#v100588) from Vienna Drosophila RNAi Center (VDRC) (Vienna, Austria) were used. To induce expression of dsRNA for *Nup58*, *P{TRiP.HMC05104}attP40* (#60110) from BDSC and *P{KK101515}VIE-260B* (#v108016) from VDRC were used. To induce of dsRNA for *Nup54*, *P{TRiP.HMC04733}attP40* (#57426) from BDSC, *P{GD14041}v42153* (#v42153) and *P{KK102105}VIE-260B* (#v103724) from VDRC were used. To induce expression of dsRNA for *emb*, *P{TRiP.HMS00991}attP2* (#34021), *P{TRiP.JF01311}attP2* (#31353) from BDSC were used. As a Gal4 driver for spermatocyte-specific expression dependent on Gal4, we used *P{bam-Gal4::VP16}* [30]. For a depletion experiment, *P{UAS-dcr2}*; *P{bam-GAL4::VP16}* was used [31]. For a maternal induction of dsRNA, we used *w*; *P{w^+mC^ = matalpha4-GAL-VP16}V2H* (#7062) as a Gal4 driver [32]. To induce a constitutively active forms of CDK1, *UASp-CDK1^T^*^14 *A*^*::VFP, UASp-CDK1^Y^*^15 *F*^*::VFP* and *UASp-CDK1^T^*^14 *AY*15 *F*^*::VFP* were used [25] (kind gifts from S. Campbell (Alverta University, Canada)). *P{Nup58-GFP}* (a kind gift from C. Lehner, Univ. Zurich, Switzerland) was used to recognize the Nup using a GFP-tag [33]. *P{Sa-GFP}* was used as a marker to determine developmental stages of premeiotic spermatocytes (S1 to S6) [17,30,34]. *twe* (#4274), *twe^k^*^08310^ (#12212) and *w*^1118^ (#3605) were obtained from Bloomington *Drosophila* Stock Centre (BDSC, Bloomington, Indiana, USA). 

All *Drosophila melanogaster* stocks were maintained on standard cornmeal food at 25 °C, as previously described [35]. Food: 7.2 g of agar, 100 g glucose, 40 g dried yeast, and 40 g of cornmeal was added into 1L water, mixed and boiled while stirring constantly. After the food media had cooled down, 5 mL of 10% parahydroxybenzonate dissolved in ethanol and 5 mL of propionic acid were added as antiseptics. Gal4-dependent expression was done at 28 °C. 

### 2.2. Transformation

pUAST-Nup62-CFLAGHA plasmid that permits expression of cDNA for Nup62 protein fused with FLAG- and HA- tags at its carboxyl terminal under the UAS sequences. The expression plasmid was selected among the BDGF Tagged ORF collection supplied from the Drosophila Genomics Resource Center (Bloomington, Indiana, USA). The purified plasmid DNA was injected into *Drosophila* embryos via PhiC31 integrase-mediated germ line transformation using *Drosophila* Embryo Injection Services of the BestGene Inc. (Chino Hills, CA, USA).

### 2.3. Quantitative Real-Time PCR (qRT-PCR) Analysis

For qRT-PCR analysis, total RNA was extracted from young adult testes using the TRIzol reagent (Thermo Fisher Scientific, Waltham, MA, USA). cDNA synthesis from the RNA was carried out using a PrimeScript II High Fidelity RT-PCR kit (Takara, Shiga, Japan) with oligo dT primers. qRT-PCR was performed using FastStart Essential DNA Green Master (Roche, Mannheim, Germany) and a LightCycler Nano (Roche, Basel, Switzerland). *RP49* was used as a normalization reference [35]. Relative mRNA levels were quantified using LightCycler Nano software version 1.0 (Roche, Basel, Switzerland). The primers used were as follows: *Nup62* (FW: 5′- TGAATTCGTTGCAGTGGATCG-3′, RV: 5′-TCTGGGAGTCTTGAATCTTGCC -3′), *Nup58* (FW: 5′-TTCACGAATGTCAGCCACGA-3′, RV: 5′-ATCGCCTTGACGGTCTCTTG-3′), *Nup54* (FW: 5′- TCTAGGTGTTGTGGAGGCTTTG-3′, RV: 5′-CGGGGTGGATTTTCAAGGTAC-3′), *emb* (FW: 5′-TCATTATGATCTCGCGCATGGC-3′, RV: 5′- TTCGCGCATGTTCTTGTACAGG-3′). Each sample was duplicated on the PCR plate, and the final results averaged three biological replicates. For the quantification, the ∆∆Ct method was used to determine the differences between target gene expression relative to the reference *Rp49* gene expression.

### 2.4. Preparation of Onion Stage Spermatids

To judge whether two consecutive meiotic divisions were properly performed, we observed nuclei in post-meiotic spermatids under phase contrast microscopy, as previously described [36,37]. A pair of testes from pharate adults or newly eclosed adult flies (0–1 day old) were dissected to isolate spermatocyte cysts in Testis buffer (183 mM KCl, 47 mM NaCl, 10 mM EDTA, pH 6.8) and covered with 18 × 18 mm-coverslip (Matsunami, Osaka, Japan) to flatten the cysts. For observation of nuclear organization in onion stage spermatids, thecysts of spermatids collected from adult testes in Testis buffer supplemented with DAPI were mildly flattened under a cover slip, and phase contrast micrographs and fluorescence images were successively acquired [36,37]. To observe fixed spermatid samples, we removed the coverslips after freezing the slides, and transferred them into 100% methanol for 5 min. Consequently, the samples were rehydrated in 1× PBS (137.0 mM NaCl, 2.7 mM KCl, 10.1 mM Na_2_HPO_4_·12H_2_O, 1.8 mM KH_2_PO_4_) for 10 min and then stained with DAPI. Under a phase contrast microscope, we observed cysts of spermatids after a completion of meiosis II, at the stage called onion stage and a slightly later stage. We counted the numbers of spermatids containing in single intact cysts; cysts consisted of 64 spermatids were generated by two consecutive meiotic divisions, while cysts consisted of 32 and 16 spermatids resulted from single meiotic divisions and from spermatid differentiation without meiotic divisions. Samples were observed with a phase-contrast microscope (Olympus, Tokyo, Japan, model: IX81). 

### 2.5. Immunofluorescence Microscopy

Testis cells were fixed according to the method as described above. The slides were permeabilized in PBST (PBS containing 0.01% Triton-X) for 10 min and blocking with 10% normal goat serum in PBS for 30 min at room temperature. Primary antibodies were used at the dilution described: monoclonal mouse anti-Cyclin B antibody (F2F4), 1:200 (Developmental Studies Hybridoma Bank (DSHB), Iowa Univ. Iowa, USA), polyclonal rabbit anti-HA (C29F4), 1:400 (Cell Signaling Technology, Danvers, MA, USA), monoclonal mouse anti-Lamin Dm0 (ADL84.12), 1:200 (DSHB, Iowa Univ. Iowa, USA), polyclonal rabbit anti-CDK1 (06-923), 1:200 (Sigma–Aldrich, St. Louis, MO, USA), polyclonal rabbit anti-phospho CDK1 at Thr^160^ (ab194868), 1:200 (Sigma–Aldrich, St. Louis, MO, USA), polyclonal rabbit anti-GFP antibody (A-6455), 1:800 (Thermo Fischer Scientific, Waltham, USA), and monoclonal mouse anti-phospho-Ser/Thr-Pro MPM-2 antibody (05-368), 1:400 (Sigma–Aldrich, St. Louis, USA). After incubating over night at 4 °C, the slides were repeatedly washed in PBS and subsequently incubated with Goat Anti-Rabbit or Anti-Mouse IgG (H + L) Alexa Fluor 488 or 594 (Thermo Fisher Scientific, Waltham, USA). After incubation for 2 h at room temperature, the slides were washed in PBS for 10 min. The samples were mounted with VECTASHIELD Mounting Medium with DAPI (Vector Laboratories, Burlingame, USA). Samples were observed with a fluorescent microscope (Olympus, Tokyo, Japan, model: IX81). Image acquisition was controlled through the Metamorph software version 7.6 (Molecular Devices). 

### 2.6. Proximity Ligation Assay (In Situ PLA) 

In situ PLA that enables detection of protein interaction within a cell was performed according to the Duolink kit method (Nacalai Inc., Kyoto, Japan). We applied the in situ PLA method to examine a close association between three sets of proteins, Emb and CycB, Emb and Nup58, and Nup62 and CycB. For the detection of complexes containing the first protein set, we used anti-HA and anti-CycB antibodies to recognize complexes containing Emb-HA and Cyclin B. We used anti-HA and anti-GFP antibodies to detect complexes containing Emb-HA and Nup58-GFP. We used anti-HA and anti-CycB antibodies to recognize complexes containing Nup62-HA and CycB. We observed a positive control of in situ PLA signals indicating association between CycB and CDK1 using the relevant antibodies. Samples were observed with a fluorescent microscope (Olympus, Tokyo, Japan, model: IX81). Image acquisition was controlled through the Metamorph software version 7.6 (Molecular Devices) and processed with ImageJ version 1.51 or Adobe Photoshop CS4.

## 3. Results

### 3.1. Depletion of the Nup62 Complex Components Constructing the Central Channel of NPC Resulted In Cell Cycle Arrest of Premeiotic Spermatocytes before Male Meiosis in Drosophila

A previous study investigated the involvement of the NPC in cell cycle progression before and during male meiosis in *Drosophila* [17]. The authors performed spermatocyte-specific RNAi experiments to knockdown Nups in the spermatocytes. They induced the spermatocyte-specific expression of dsRNAs against 21 Nup mRNAs using the *bam-Gal4* driver and determined whether meiotic phenotypes appeared in post-meiotic cells called spermatids after the completion of meiosis II. Their results showed that meiotic cells were absent in testes with spermatocyte-specific expression of dsRNAs against the mRNAs for *Nup54, Nup58, Nup62, Nup154*, and *Rae1*. Nup154 constructs the inner rings linking the central channel of NPC. Rae1 is involved in mRNA export through the NPC. The observation that depletion of these two Nups resulted in the loss of meiotic cells was consistent with previously published results that meiosis did not occur in *Nup154* mutant and *Rae1*-depleted testes [38,39]. On the other hand, the other three Nups of the Nup62 complex constructs the central channel in NPC. These results encouraged us to investigate the role of the Nup62 complex in the initiation of meiotic division in *Drosophila* males. 

**Figure 1:**
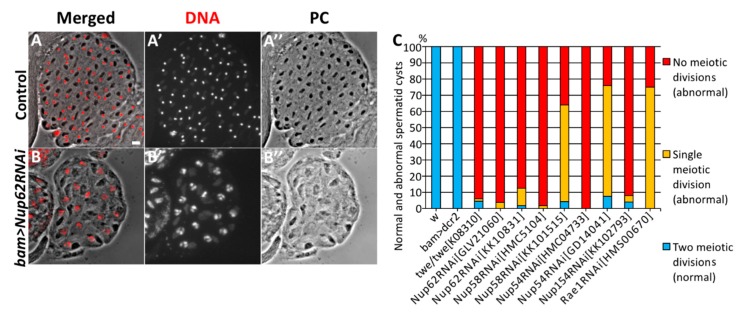
Abnormal cysts consisting 25% and 50% of the normal spermatids generated from spermatocytes depleted of Nup62 complex components. (**A**,**B**) Phase contrast micrographs of a single intact cyst composed of spermatids at the onion stage and slightly later stage. (**A**) A normal spermatid cyst contained 64 cells after the completion of meiosis II, which were generated by two consecutive meiotic divisions of 16 premeiotic spermatocytes. Every cell possesses a single nucleus (stained with DAPI) and a single Nebenkern, which is a mitochondrial derivative. (**B**) An intact cyst consisting of only 16 spermatids. Each spermatid possesses a larger nucleus and a single Nebenkern. The presence of the abnormal post-meiotic cyst suggests that neither of the meiotic divisions had occurred in the relevant cyst consisting of 16 premeiotic spermatocytes. Nuclei stained with DAPI are colored in red (**A**,**B**) and white (**A’**,**B’**). Black round-shaped structures adjacent to nuclei in the spermatids correspond to Nebenkern, a mitochondrial derivative (**A**,**A”**,**B**,**B”**). Bar: 10 μm. (**C**) Frequencies of abnormal spermatid cysts, consisting of 64 (normal), 32 (abnormal, generated by either of meiotic divisions), or 16 cells, which were generated without any meiotic divisions (abnormal). These spermatids were prepared from testes of normal males (*w*), *twe* mutant (*twe/twe^k^*^08310^) males, control males for depletion experiments (*bam>dcr2*), males with depletion of three components of the Nup62 complex (*bam*>*dcr2 Nup62RNAi^GLV^*^21060^, *bam*>*dcr2 Nup62RNAi^KK^*^10831^, *bam*>*dcr2 Nup58RNAi^HMC^*^5104^, *bam*>*dcr2 Nup58RNAi^KK^*^101515^, *bam*>*dcr2 Nup54RNAi^HMC^*^04733^, *bam*>*dcr2 Nup54RNAi^GD^*^14041^), and males with depletion of Nup154 (*bam*>*dcr2 Nup54RNAi^KK^*^102793^) and Rae1 (*bam*>*dcr2 rae1RNAi^HMS^*^00670^).

First, we induced the spermatocyte-specific expression of dsRNAs against the mRNAs of *Nup62, Nup58*, and *Nup54* using two different UAS-RNAi stocks per gene. Our qRT-PCR analysis confirmed that ectopic expression of these dsRNAs efficiently depleted the relevant mRNAs to <10% of the control level (*bam > dcr2*) in the following males: *bam*>*dcr2 Nup62RNAi^GLV^*^20160^, *bam*>*dcr2 Nup62RNAi^KK^*^10831^, *bam*>*dcr2 Nup54RNAi^HMC^*^04733^, *bam*>*dcr2 Nup54RNAi^GD^*^14041^, *bam*>*dcr2 Nup58RNAi^HMC^*^5104^, and *bam*>*dcr2 Nup58RNAi^KK^*^101515^ (Supplementary Figure S1A–C). Next, we assessed the cysts of spermatids at the onion stage immediately after the completion of meiosis II. Sixteen pre-meiotic spermatocytes consisting of a cell unit called the cyst undergo two consecutive meiotic divisions synchronously. As a result of the 1st meiotic division, a cyst consisting of 32 secondary spermatocytes is produced. Subsequently, a cyst consisting of 64 spermatids is produced in the 2nd meiotic division. Each of the post-meiotic cells in a spermatid cyst at the onion stage contains a single haploid nucleus and a single round-shaped structure called Nebenkern, a large mitochondrial derivative (Figure 1A). In the testes of *twe* mutants (*twe/twe^k^*^08310^) that could not initiate meiotic divisions, most spermatid cysts consisted of only 16 cells (Figure 1C). Even in the absence of meiotic divisions, spermatocytes can differentiate into spermatids. Thus, we can estimate the number of times meiotic divisions have occurred by counting the numbers of post-meiotic cells in a spermatid cyst. For example, in testes with spermatocyte-specific depletion of the *Nup62* mRNA (*bam>Nup62RNAi^GLV^*^21060^), we observed that 95% of the spermatid cysts at the onion stage consisted of 16 cells (Figure 1B,C), while all the normal spermatid cysts contained 64 post-meiotic cells at the same stage (Figure 1A). Every cell in the abnormal cysts possessed a nucleus larger in diameter than the nuclei of normal spermatids at this stage (Figure 1B). This meiotic phenotype appeared in spermatids from the *Nup62*-depleted spermatocytes, which is similar to that observed in *twe* mutants (Figure 1C). Therefore, we concluded that the spermatid cysts consisting of 16 and 32 cells resulted from a failure of both or either of the two meiotic divisions, respectively. This phenotype of the spermatid cysts suggests that meiotic divisions did not occur in the *Nup62*-depleted spermatocytes. We have confirmed that this meiotic phenotype appeared as a consequence of spermatocyte-specific RNAi experiments using different *UAS-Nup62* RNAi (*bam>dcr2 Nup62RNAi^GLV^*^21060^ and *bam>dcr2 Nup62RNAi^KK^*^10831^) (Figure 1C). We next investigated whether this meiotic phenotype also appears in *Nup54* and *Nup58*-depleted testes. We generated males carrying *bam-Gal4* and *UAS-Nup54RNAi* or *UAS-Nup58RNAi* using two independent *UAS-RNAi* stocks. Separately, we confirmed that the relevant mRNAs were efficiently depleted in the testes (Supplementary Figure S1A–C). We observed the same phenotype in 96.2% of the spermatid cysts in *bam*>*Nup62RNAi^GLV^*^21060^ (n = 51/53), in 98.3% of the cysts in *bam*>*Nup58RNAi^HMC^*^5104^ (n = 57/58), and in 100% of the cysts in *bam*>*Nup54RNAi^HMC^*^04733^ (n = 50/50) (Figure 1C). We also confirmed that unlike in normal cells, Nup54, another member of the Nup62 complex, was no longer localized on the nuclear envelope of *Nup62*-depleted cells (Supplementary Figure S2(A”’,B”’)). This indicated that *Nup62* depletion inhibited the formation of the Nup62 complex containing Nup54. From these observations, we concluded that a depletion of the Nup62 complex resulted in the inhibition of meiotic initiation in *Drosophila* males.

Furthermore, we investigated whether depletion of *Nup62* also affects mitosis of spermatogonia. In *Drosophila* spermatogenesis, a gonialblast derived from a germline stem cell undergoes four rounds of mitosis. Consequently, a cyst consisting of 16 spermatocytes is generated. For the efficient depletion of *Nup62* mRNA in spermatogonia, we used the same *UAS-RNAi* stocks and a *nos-Gal4* driver that allows us to induce gene expression in both spermatogonia and spermatocytes [40]. We counted the number of cells in a spermatocyte cyst, which were generated by mitoses of the spermatogonium at the S2b stage, an earlier growth stage (Supplementary Figure S3A). The cells in this stage show a unique distribution of organelles, such as a polar distribution of the nucleus and mitochondrial aggregates adjoining the nucleus. Thus, we were easily able to recognize the spermatocyte cysts at this stage. We confirmed that every spermatocyte cyst in the S2b stage contained 16 cells in the control testes (*nos>dcr2*) (Supplementary Figure S3A) (n = 11 cysts). Similarly, we examined 60 premeiotic cysts and demonstrated that all the spermatocyte cysts in *nos>dcr2 Nup62RNAi ^GLV^*^21060^ males also consisted of 16 cells (Supplementary Figure S3C), as observed in the control testes. In this case, we confirmed that Nup62 was efficiently depleted in the cells, because we observed spermatid cysts consisting of 16 cells at high frequency (88.2%, 45/51 cysts examined). In control testes, every control cyst contained 64 spermatids (*nos*>*dcr2*, n = 10) (Supplementary Figure S3B,D). These observations strongly suggested that depletion of *Nup62* did not affect mitosis of spermatogonia, unlike *Drosophila* male meiosis and mitosis in mammalian cells [40]. 

### 3.2. CDK1 Was Not Activated in Nup62-Depleted Premeiotic Spermatocytes, Even at the Last Stage before Meiotic Initiation

**Figure 2:**
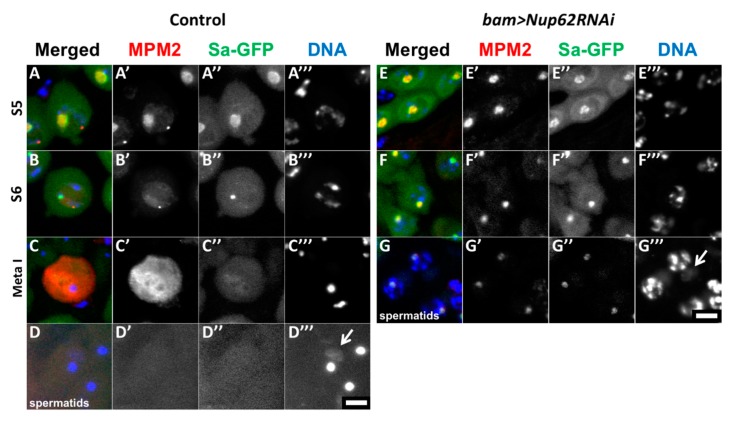
Depletion of Nup62 complex components resulted in inhibition of CDK1 activation. (**A**–**G**) Immunostaining of spermatocytes at later stages with anti-MPM2 antibody, which detects protein phosphorylation by CDK1. Anti-MPM2 immunostaining (red in (**A**–**G**), white in (**A’**–**G’**)), Sa-GFP fluorescence as a marker to determine the developmental stages of spermatocytes (green in A-G, white in (**A”**–**G”**)), DAPI staining (blue in (A–G–, white in (**A’’’**–**G’’’**)). (**A**–**D**) Pre-meiotic spermatocytes (**A**–**C**) and post-meiotic cells of spermatids (**D**) in normal control testes (*bam>dcr2*). (**E**–**G**) Pre-meiotic spermatocytes (**E**,**F**) and spermatids (**G**) in testes harboring spermatocyte depleted of Nup62 (*bam>dcr2 Nup62RNAi*). (**A**,**E**) Premeiotic spermatocytes in growth stage S5, in which Sa-GFP on nucleoli has a large round shape. (**B**,**F**) Premeiotic spermatocytes in the S6 stage, the last stage of the growth phase, and immediately before meiotic initiation. The Sa-GFP signal diminished as nucleoli are discomposed at the stage. The stages of the spermatocytes in the growth phase were assessed based on the cellular distribution of Sa-GFP. (**C**) A primary spermatocyte at metaphase I showing distinctive immunostaining signal with MPM2 antibody. Spermatocytes showing strong MPM2 immunostaining signal as shown in (**C**) were not observed in the depleted testes. (**D**) Normal spermatids with a single nucleus and a single Nebenkern (arrow). (**G**) Spermatid formed without meiotic divisions in the depleted testes. These cells possess condensed chromosomes in their nuclei with a larger Nebenkern (arrow). Bar: 10 μm.

To understand why a depletion of *Nup62* resulted in the inhibition of meiotic initiation, we investigated whether CDK1, a protein kinase essential for initiation of M-phase, is activated in the depleted testes (*bam>dcr2 Nup62RNAi*). We performed immunostaining with the MPM2 antibody, which can recognize proteins phosphorylated by CDK1. In control testes (*bam>dcr2*), the MPM2 signal co-localized with Sa-GFP (Figure 2(A’,A”)) in premeiotic spermatocytes at the S5 stage, in which the Sa-GFP signal can be seen in the round-shaped nucleolus. With the nucleolus collapsing, along with phosphorylation of nucleolar proteins by CDK1 [41], the MPM2 foci became smaller and the fluorescence became less intense (Figure 2(B,B’)). The strongest MPM2 immunofluorescence was observed over the cells when CDK1 was fully activated in the beginning of prophase I (Figure 2(C,C’)). Sa-GFP foci were no longer detected at this stage (Figure 2(C”)). Similarly, we observed distinctive MPM2 signal on the nucleoli in the S5 stage in the *Nup62*-depleted spermatocytes (Figure 2(E’)) and reduced MPM2 signal at a later stage (Figure 2(F’)). However, we never observed spermatocytes with remarkable MPM2 immunofluorescence similar to that observed in control testes (Figure 2C). Hence, we concluded that the cell cycle of spermatocytes depleted of *Nup62* (*bam>dcr2 Nup62RNAi*) was arrested before prometaphase I, in which CDK1 is fully activated in the cells. These immunostaining data indicated that CDK1 was not activated in the *Nup62*-depleted cells. In *Nup62*-depleted testes, spermatids possessing larger nuclei with condensed chromosomes and residual weak MPM2 signal were produced without any meiotic divisions (Figure 2G), whereas spermatids with nuclei in which chromatin was distributed homogeneously were produced by two consecutive meiotic divisions in control males (*bam>dcr2*) (Figure 2D). 

### 3.3. Phosphorylation of CDK1 at Thr^161^ Was Observed in the Nup62-Depleted Spermatocytes before Meiosis 

**Figure 3:**
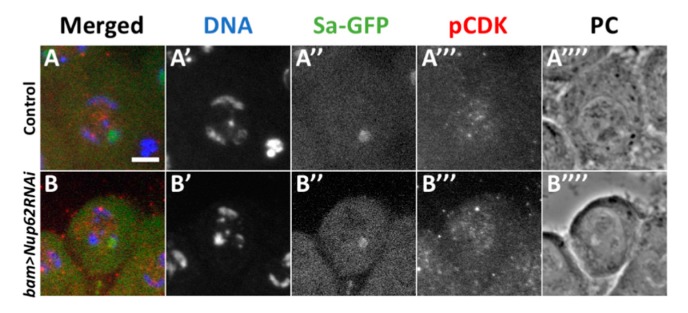
Normal phosphorylation of CDK1 at Thr^161^ was observed in premeiotic spermatocytes depleted of *Nup62*. (**A**,**B**) Immunostaining of premeiotic spermatocytes in S5 to S6 stages with an antibody recognizing a phosphorylated form of CDK1 at Thr^161^. (**A**) A control spermatocyte (*bam>dcr2*), (**B**) premeiotic spermatocytes at the same stage with spermatocyte-specific depletion of *Nup62* (*bam*>*dcr2 Nup62RNAi^KK^*^106754^). (**A’**,**B’**) DAPI staining. (**A”**,**B”**) Fluorescence of Sa-GFP for staging of spermatocytes (green in (**A**) and (**B**), white in (**A”**) and (**B”**)). Immunostaining to detect phosphorylation of CDK1 at Thr^161^ (pCDK) (red in (**A** and (**B**), white in (**A”’**) and (**B”’**)). Phase contrast micrograph (PC) to observe cell margin and nucleolus. Bar; 10 µm.

Next, we investigated why CDK1 was not activated in spermatocytes depleted of *Nup62*. Three essential modifications are required for the activation of the complex consisting of CDK1 and M-phase cyclin, namely, accumulation of CycB, a positive regulator of CDK1, phosphorylation of CDK1 at Thr^161^ by CDK-activating kinase (CAK), and dephosphorylation of CDK1 at Thr^14^ and Tyr^15^ by a phosphatase encoded by *twe*. First, we investigated whether CDK is phosphorylated at the Thr^161^ residue. In control premeiotic spermatocytes in the S5 stage, we observed immunofluorescence with a specific antibody that recognizes CDK1 phosphorylated at Thr^161^ (Figure 3A). In the *Nup62*-depleted premeiotic spermatocytes, the immunofluorescence signal was indistinguishable from that in normal cells (Figure 3B). These immunostaining data indicated that phosphorylation of CDK by CAK occurred in the depleted spermatocytes. 

### 3.4. Cell Cycle Arrest of the Nup62-Depleted Cells Was not Rescued by Expressing Constitutively Active CDK1, but Was Rescued by the Over-Expression of CycB 

**Figure 4:**
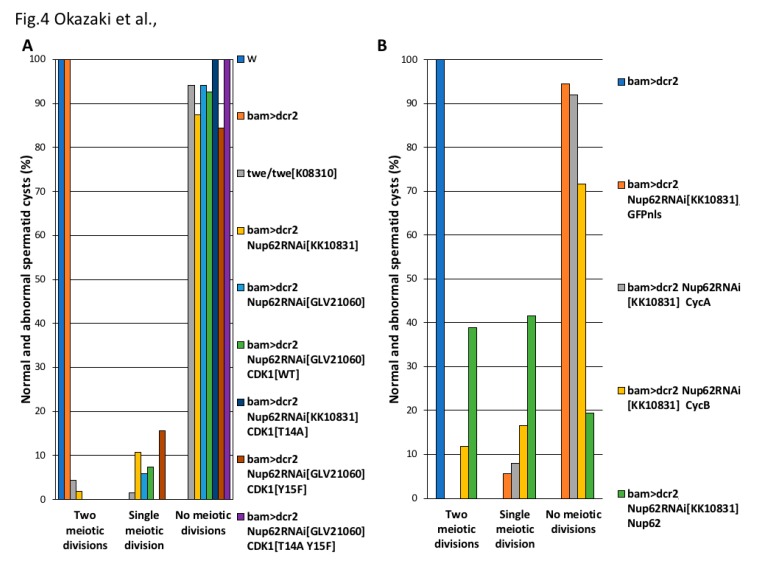
Effect of ectopic expression of constitutively active CDK1 and that of M-phase cyclin on the cell cycle arrest of the *Nup62-*depleted premeiotic cells. (**A**,**B**) Frequencies of post-meiotic cysts consisting of 16, 32, and 64 spermatids, generated from no meiotic divisions (abnormal), single meiotic division (abnormal), and consecutive two meiotic divisions (normal), respectively. (**A**) Spermatid cysts in testes with spermatocyte-specific depletion of *Nup62* and simultaneous expression of constitutively active CDK1. Blue bar, *w* (n = 56 flies); orange bar, *bam>dcr2* (n = 52 flies); light gray bars, *twe/twe^k^*^08310^ (n = 68); yellow bars, *bam>dcr2, Nup62RNAi^KK^*^10831^ (n = 56); light blue bars, *bam>dcr2 Nup62RNAi^GLV^*^21060^ (n = 68); green bar, *bam>dcr2 Nup62RNAi^GLV^*^21060^, *CDK1^WT^* (n = 27); dark blue bar, *bam>dcr2 Nup62RNAi^KK^*^10831^ *CDK1^T^*^14 *A*^ (n = 43); brown bar, *bam>dcr2 Nup62RNAi^GLV^*^21060^ *CDK1^Y^*^15 *F*^ (n = 32); purple bar, *bam>dcr2 Nup62RNAi^GLV^*^21060^ *CDK1^T^*^14 *A Y*15 *F*^ (n = 43). (**B**) Spermatid cysts in testes harboring spermatocyte-specific depletion of *Nup62* and simultaneous expression of M-phase cyclins. Blue bar, *bam>dcr2* (n = 52 flies); orange bars, *bam>dcr2, Nup62RNAi^KK^*^10831^ *GFPnls* (n = 36); light gray bars, *bam>dcr2 Nup62RNAi^KK^*^10831^ *CycA* (n = 133); yellow bar, *bam>dcr2 Nup62RNAi^KK^*^10831^ *CycB* (n = 194); green bar, *bam>dcr2 Nup62RNAi^KK^*^10831^ *Nup62* (n = 36). Note that only CycB, but neither ectopic expression of constitutively active CDK1 or CycA, can rescue from the cell cycle arrest.

CDK1 activity is negatively regulated by phosphorylation of the Thr^14^ and Tyr^15^ residues before the initiation of the M-phase. Hence, de-phosphorylation of CDK1 at these residues is essential for its activation. We verified whether de-phosphorylation of CDK1 at these residues occurred in pre-meiotic spermatocytes depleted of *Nup62*. As specific antibodies that can recognize a *Drosophila* CDK1 phosphorylated at these sites were not available, we investigated whether expression of the constitutively active forms of CDK1 can rescue cells from cell cycle arrest. CDK1^T14A^, CDK1^Y15F^, and CDK1^T14A Y15F^ are mutant forms of CDK1 that are not phosphorylated. We analyzed whether the ectopic expression of these CDK1 mutants can rescue *Nup62*-depleted spermatocytes from cell cycle arrest (Figure 4A). In *Nup62*-depleted spermatocytes, we induced the expression of a normal CDK1 or its constitutively active forms, CDK1^T14A^, CDK1^Y15F^, or CDK1^T14A,Y15F^. After confirming the production of these four types of YFP-tagged CDK1 using an anti-GFP antibody (data not shown), we investigated whether any spermatid cysts consisting of 32 cells and 64 cells existed in the testes. In *Nup62*-depleted testes (*bam>dcr2 Nup62RNAi^GLV^*^21060^), 94% of the spermatid cysts consisted of 16 cells (64/68 cysts examined) and the remaining 6% consisted of 32 cells (4/68). The simultaneous expression of a normal CDK1 in the depleted testes did not affect the meiotic phenotype; 93% of the spermatid cysts consisted of 16 cells (25/27 cysts) and 7% consisted of 32 cells (2/27) in *bam>dcr2 Nup62^GLV^*^21060^ *CDK1^WT^*. Similarly, in testes showing the ectopic expression of three constitutively active mutants of CDK1, the frequencies of spermatid cysts containing 16 cells did not decrease. All spermatid cysts in *bam>dcr2 Nup62^KK^*^10831^ *CDK1^T^*^14 *A*^ (n = 43) and the cysts in *bam>dcr2 Nup62^GLV^*^21060^ *CDK1^T^*^14 *A Y*15 *F*^ (n = 27) consisted of only 16 spermatids, although the frequency was slightly lower in *bam>dcr2 Nup62^GLV^*^21060^ *CDK1^Y^*^15 *F*^ (n = 32) (Figure 4A). Therefore, when considered together with the aforementioned results of CDK1 phosphorylation at Thr^161^, we concluded that inactivation of CDK1 in the *Nup62*-depleted spermatocytes was not responsible for the absence of CDK1 modification. 

Therefore, we next investigated whether the spermatocyte-specific over-expression of M-phase cyclins essential for CDK1 activity can suppress the cell cycle arrest. In premeiotic spermatocytes depleted of *Nup62* (*bam>dcr2 Nup62RNAi^KK^*^10831^), 94% of the spermatid cysts consisted of 16 cells (34/36 cysts examined), and 6% was composed of 32 cells (2/36). We over-expressed two types of M-phase cyclins, CycA, and CycB, in *Nup62*-depleted testes of *Drosophila* (Figure 4B). The over-expression of CycA in the depleted spermatocytes could not rescue the cell cycle arrest before meiotic initiation; 92% of the spermatid cysts consisted of 16 cells (123/133 cysts examined) and 8% was composed of 32 cells (10/133). In contrast, the over-expression of CycB in the *Nup62*-depleted cells resulted in partial rescue of the cell cycle arrest phenotype. Twelve percent of the spermatid cysts consisted of 64 cells (23/194), although 72% of the spermatid cysts still consisted of 16 cells (139/194 cysts examined) and 16% was composed of 32 cells (32/194). These genetic data suggested that lack of CDK1 activation before meiotic initiation in the *Nup62*-depleted spermatocytes was because of expression or distribution of CycB.

### 3.5. Depletion of Nup62 in Premeiotic Spermatocytes Resulted in the Accumulation of CycB in Nuclei before the Initiation of Drosophila Male Meiosis

**Figure 5:**
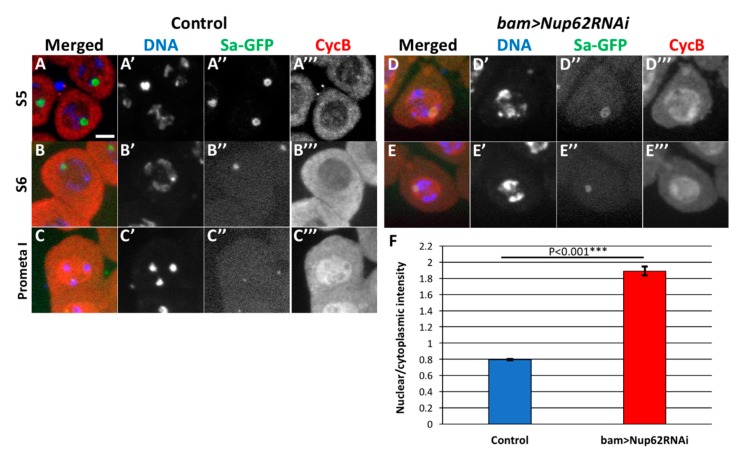
Depletion of Nup62 complex components compromised intracellular localization of CycB before meiotic division I. (**A**–**E**) Immunostaining of pre-meiotic spermatocytes at later growth stages with anti-CycB antibody. (**A**) Control primary spermatocytes in premeiotic stage S5, in which condensing chromosomes and distinctive Sa-GFP signal on nucleoli were observed. CycB accumulated in the cytoplasm but not in the nucleus in normal spermatocytes. (**B**) Control primary spermatocytes in the S6 stage, in which Sa-GFP was localized on diminishing nucleoli. (**C**) A prophase I cell, in which Sa-GFP foci has almost disappeared. Note that the accumulation of CycB in the nucleus was initiated at the onset of meiosis. (**D**,**E**) Nuclear accumulation of CycB in the latest stage of premeiotic spermatocytes depleted of *Nup62* (*bam>dcr2 Nup62RNAi^GLV^*^21060^). (**D**) CycB was localized in both nucleus and cytoplasm of the S5 cells that harbors condensing chromosomes and a distinctive Sa-GFP signal on the nucleolus. (**E**) Accumulation of CycB in the nuclei of the latest spermatocytes in the *Nup62*-depleted testis. The late S5 to S6 cells contained condensed chromosomes and the remaining Sa-GFP signals on the nucleoli. Blue; DAPI staining (white in (**A’**–**E’**)); green, Sa-GFP fluorescence for determining the developmental stages of premeiotic spermatocytes (white in (**A”**–**E”**)); red, immunostaining with anti-CycB antibody (white in (**A”’**–**E”’**)). Bar, 10 μm. (**F**) Ratio of anti-CycB immunofluorescence intensity in nuclei over that in the cytoplasm. Blue bar, normal control spermatocytes (*bam>dcr2*, n = 173); red bar, *Nup62*-depleted spermatocytes (*bam>dcr2 Nup62RNAi^GLV^*^21060^, n = 177). *** *p* < 0.001, Student’s *t*-test. Bar; 10 μm.

To understand whether the expression of CycB and/or its cellular localization is perturbed in the *Nup62*-depleted premeiotic spermatocytes, we performed immunostaining of the spermatocytes with anti-CycB antibody (Figure 5). CycB accumulated in the cytoplasm in normal spermatocytes in the S5 stage with distinct Sa-GFP foci on nucleoli (Figure 5(A,A”’)). Judging from previous studies using mammalian cells, this is a consequence of nuclear export of the CycB. Subsequently, the highest amount of the protein was observed in the cytoplasm in the S6 stage, in which the size of the Sa-GFP foci had diminished (Figure 5B). CycB became to be accumulated in the nuclei after the Sa foci disappeared at prophase I (Figure 5C). We compared the intracellular localization of CycB in normal spermatocytes with that in the *Nup62*-depleted spermatocytes in the same stages, as was evident from the Sa-GFP signal. Lesser immunofluorescence was observed in the cytoplasm of the *Nup62*-depleted spermatocytes in the S5 stage than in normal spermatocytes in the same stage. Accumulation of CycB into the nucleus occurs in premeiotic spermatocytes depleted of *Nup62* in the earlier S5 stage, as was evident from the remaining apparent Sa-GFP foci (Figure 5D). The nuclear accumulation of CycB was observed in premeiotic spermatocytes in the S6 stage, in which decrease in Sa-GFP foci was observed (Figure 5E,F). Large amounts of CycB did not accumulate in prophase I spermatocytes in the depleted testes.

### 3.6. Persistent Nuclear Accumulation of CycB Was also Observed in Spermatocytes Depleted of an Exportin Orthologue Encoded by Emb 

**Figure 6:**
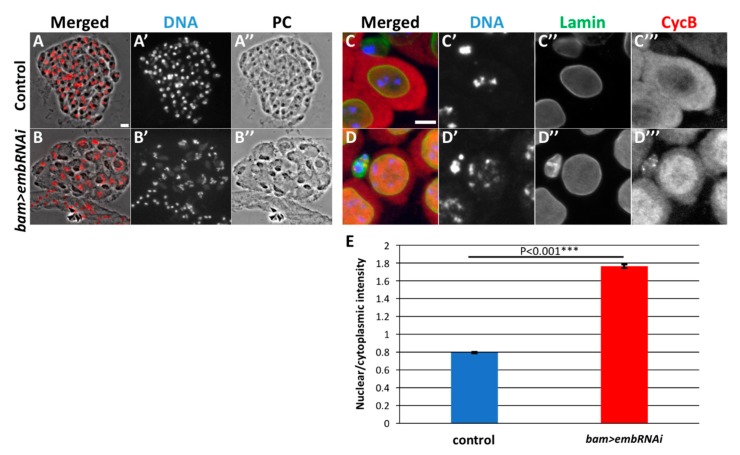
Depletion of *emb* resulted in meiotic cell cycle defect and abnormal CycB distribution, as observed in testes depleted of the Nup62 complex. (**A**,**B**) Phase contrast and fluorescence micrographs of an intact cyst consisting of spermatids at the onion stage and slightly later stage. Fluorescence of DAPI staining is in red (**A**,**B**) and white (**A’**,**B’**). PC; Phase contrast micrograph (**A”**,**B”**). (**A**) A complete cyst consisted of 64 spermatids in a control testis (*bam>dcr2*). (**B**) A complete cyst consisted of 16 spermatids in *emb*-depleted testis. Each of the cells possesses a single larger nucleus containing three sets of condensed chromosomes and Nebenkerns. (**C**,**D**) Immunostaining of premeiotic spermatocytes at later stage of growth phase with anti-CycB (red in (**C**,**D**), white in (**C”**,**D”**)) and anti-Lamin Dm0 (green in (**C**,**D**), white in (**C’”**,**D”’**)) antibodies. DAPI staining, blue in (**C**,**D**), white in (**C’**, **D’**). (**C**) Control premeiotic spermatocytes in the S5 stage, with condensed chromatin and intact nuclear envelope. Note that CycB is localized in the cytoplasm rather than in the nuclei. (**D**) In *emb*-depleted spermatocytes at the equivalent stage (*bam>dcr2 embRNAi^HMS^*^00991^), having intact nuclear envelope (**D”**). Precocious accumulation of CycB was observed (**D”’**). Bar: 10 μm. These phenotypes are similar to those observed in testes depleted of the Nup62 complex. (**E**) Ratio of the anti-CycB immunofluorescence intensities in the nuclei and cytoplasm. *** *p* < 0.001, Student’s *t*-test.

We demonstrated that CycB enters the nucleus precociously before sufficient amount of the protein accumulates in the cytoplasm in the *Nup62*-depleted spermatocytes. CycB can be easily transported into the nucleus before mitosis. Reports show that one of the exportins, CRM1, is involved in the cytoplasmic export of the CycB that has temporally been imported before the M-phase in Hela cells (see Introduction). Hence, we investigated whether a CRM1 orthologue, Emb is involved in the nuclear export of CycB before meiotic initiation in *Drosophila* males. First, we confirmed whether that ectopic expression of the dsRNA against *emb* mRNA using UAS-*emb RNAi^HMS^*^00991^ is able to deplete the endogenous mRNA level to 17% of the control levels (Supplementary Figure S1D). We next investigated whether Emb is involved in the nuclear export of CycB and regulation of meiotic initiation in males. In testes harboring spermatocyte-specific depletion of *emb* (*bam*>*dcr2 embRNAi^HMS^*^0091^ or *bam*>*dcr2 embRNAi^JF^*^01311^), we observed many premeiotic spermatocytes but no meiotic cells; no meiotic cysts were scored in *bam*>*dcr2 embRNAi^HMS^*^0091^ (n = 29 flies). No meiotic cysts were recognized in the testes of *bam*>*dcr2 embRNAi^JF^*^01311^ (n = 25 flies). We observed five onion stage spermatid cysts consisting of 16 cells in the Emb-depleted testes (*bam*>*dcr2 embRNAi^JF^*^01311^) (Figure 6B). These phenotypes are reminiscent of those in *Nup62*-depleted testes (Figure 1B) and indicate that spermatocytes depleted of *emb* cannot initiate meiotic divisions. 

Hence, we performed immunostaining of the *emb*-depleted premeiotic spermatocytes with anti-CycB antibody (Figure 6C,D). Anti-CycB fluorescence was higher in the cytoplasm than in the nucleus in control spermatocytes (*bam>dcr2*) until the S5 stage before meiosis (Figure 6C). The ratio of the immunofluorescence intensity of CycB in the nucleus to that in the cytoplasm was calculated to be 0.8 in normal spermatocytes (Figure 6E). In contrast, a considerable amount of the protein had already entered the nuclei of the Emb-depleted spermatocytes (*bam*>*embRNAi^HMS^*^00991^) (Figure 6D). The average ratio of the anti-CycB immunofluorescence in the nucleus to that in the cytoplasm in the e*mb*-depleted cells was 1.8 (Figure 6E), which was significantly higher than that in the control (*bam>dcr2*). Consequently, sufficient cytoplasmic accumulation of CycB was not observed (Figure 6(D”’)). Taken together, Emb is also required for the export of CycB that has temporally entered the nucleus temporally before meiosis. 

### 3.7. In Situ Proximity Ligation Assay (PLA) Demonstrated the Presence of Protein Complexes Containing Emb and CycB, Emb and Nup58, and Nup62 and CycB in the Nuclei of Normal Spermatocytes before Meiosis

**Figure 7:**
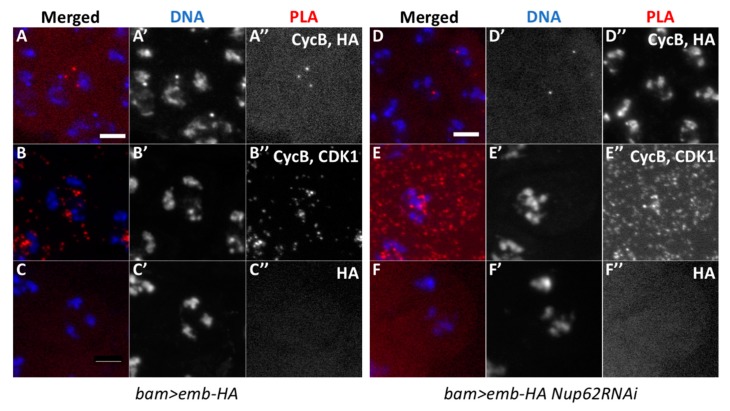
Proximity ligation assay (in situ PLA) showing close association between Emb and CycB in normal premeiotic spermatocytes and *Nup62*-depleted cells before initiation of meiosis I. (**A**–**F**) In situ PLA of premeiotic spermatocytes for detecting protein complexes. The PLA foci are in red (**A**–**F**) and white (**A”**–**F”**). (**A**) The PLA foci with anti-CycB and anti-HA antibodies indicate the presence of protein complexes containing CycB and Emb-HA in normal spermatocytes expressing Emb-HA. (**B**) A positive control of the assay with anti-CycB and anti-CDK1 antibodies. (**C**) A negative control for in situ PLA with anti-HA antibody alone (0.31%, n = 318). (**D**–**F**) In situ PLA of the spermatocytes with Emb-HA expression and depletion of *Nup62.* (**D**) Assay with anti-CycB and anti-HA antibodies; 20.3% of the *Nup62*-depleted cells contained the PLA foci, indicating presence of protein complexes containing CycB and Emb (n = 320). (**E**) A positive control experiment with anti-CycB and anti-CDK1 antibodies (99.4%, n = 167). (**F**) A negative control experiment with only anti-CycB antibody (1.1%, n = 184). Blue; DAPI staining.

The nuclear export of CycB to the cytoplasm is mediated by the exportin CRM1, which binds to the nuclear export signal (NES) of the M-phase cyclin. Here, we investigated whether a *Drosophila* CRM1 orthologue, Emb, is closely associated with CycB before initiation of meiosis using in situ PLA. We induced the expression of HA-tagged Emb in spermatocytes using *bam*-GAL4. Subsequently, we performed anti-HA immunostaining to confirm the expression of Emb-HA and analyzed its intracellular localization. The protein was mainly localized in association with the nuclei of spermatocytes (data not shown). We performed the simultaneous immunostaining of the premeiotic spermatocytes with anti-CycB antibody and observed a small amount of CycB in the nuclei of spermatocytes in the S5 stage before meiosis (Figure 5A). Next, we performed in situ PLA to detect protein complexes containing CycB and Emb in the spermatocytes expressing Emb-HA. First, we confirmed the specificity of the in situ PLA experiments, by showing the presence of the CycB-CDK1 complex with anti-CycB and anti-CDK1 antibodies in mature spermatocytes before meiosis I (Figure 7B). The PLA foci appeared in 54.6% of the spermatocytes (n = 637 examined). Cells containing PLA foci were not observed using HA antibody alone (n = 318 examined, Figure 7C). Subsequently, we performed the in situ PLA experiments to observe a complex containing CycB and Emb. We observed PLA foci in 25.4% of the premeiotic spermatocytes in the S5 stage expressing Emb-HA (n = 634 cells) (Figure 7A). These data strongly suggested that CycB is closely associated with Emb, showing that these two proteins existed at least within 40 nm before meiotic initiation. Furthermore, we investigated whether the formation and/or maintenance of the temporal protein complex containing CycB and Emb was dependent on Nup62. We performed the same in situ PLA experiments in spermatocytes depleted of *Nup62*. As a result, we observed the PLA signals at a frequency equivalent to that in control cells (20.3%, n = 320) (Figure 7D). 

**Figure 8:**
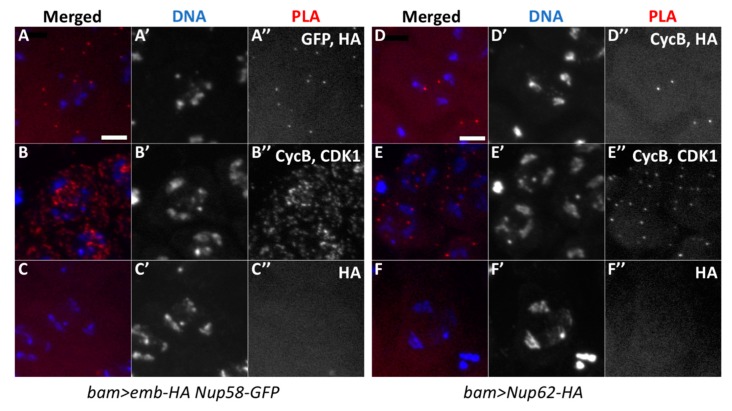
In situ PLA to detect protein complexes containing Emb and Nup58 of the Nup62 complex, and complexes containing Nup62 and CycB. (**A**) In situ PLA for detection of complexes containing Emb and Nup58. The cells expressing Emb-HA and Nup58-EGFP were analyzed using anti-HA and anti-GFP antibodies. (**B**) The assay with anti-CycB and anti-CDK1 antibodies as the positive control (48.5%, n = 429), (**C**) The assay with only anti-HA antibody as the negative control (1.4%, n = 287). (**D**–**F**) In situ PLA to detect complexes containing Nup62 and CycB. (**D**) Assay of spermatocytes expressing Nup62-HA with anti-HA and anti-CycB antibodies. (**E**) Anti-CycB and anti-CDK1 antibodies were used as positive controls (87.2%, n = 358); (**F**) with only anti-HA antibody as the negative control (1.3%, n = 378). The PLA signal is in red (white in (**A”**–**F”**)) and DAPI in blue (white in (**A’**–**F’**)).

Using similar in situ PLA experiments, we also detected PLA foci of protein complexes containing Emb and Nup58 in spermatocytes simultaneously expressing Nup58-GFP and Emb-HA (25.2%, n = 262) (Figure 8A). We detected signals corresponding to the complexes containing CycB and Nup62 in spermatocytes expressing Nup62-HA (20.1%, n = 448) (Figure 8D). Based on these data, we concluded that CycB, Emb, and Nup62 are closely associated in premeiotic spermatocytes before meiotic initiation. The complexes containing CycB and Emb were formed independent of the Nup62 complex. 

## 4. Discussion

### 4.1. Selective Nuclear Export of CycB, Essential for the CDK Full Activation before the Onset of Drosophila Male Meiosis, Is Mediated by Nup62 Complex in the Central Channel of NPC 

The CDK-M phase cyclin complex plays a critical role in triggering mitotic and meiotic cell divisions in eukaryotes. The kinase complex is activated in a step-wise manner, changing its cellular localization between the cytoplasm and the nucleus. This spatial regulation of the most important kinase for cell cycle regulation is shared among eukaryotes [42,43,44]. In this study, we demonstrated that CDK1 was not activated before initiation of meiosis I in the *Nup62*-depleted spermatocytes. A considerable amount of CycB was still present in the nucleus of the *Nup62*-depleted cells before meiosis. The intracellular localization of CycB-CDK1 is dependent on a regulatory subunit, CycB [28,29,45]. Previous studies on mammalian cells, as well as *Drosophila* embryos, revealed that Tyr^14^ and Thr^15^ of CDK1 are temporally phosphorylated by Wee1 and/or Myt1. Phosphorylation of these residues suppresses the kinase activity of the CDK1 [22,23,24,25,46]. Then, another phosphorylation of CDK1 at Thr^161^ by CAK present in the nucleus occurs. This phosphorylation is one of the essential conditions for the activation of the kinase [23,24,25,47,48]. In *Drosophila*, the CAK made of Cyclin H and CDK7 is also localized in the nucleus in early embryos [49]. In the *Nup62*-depleted premeiotic spermatocytes, this phosphorylation occurred, as observed in control cells. This is consistent with the interpretation that the phosphorylation of the CycB-CDK1 or free CDK1, if any, can be normally performed in the nucleus by the CAK in the depleted cells. Subsequently, this phosphorylated form of CDK1 with CycB is re-accumulated in the cytoplasm. This is an essential step before the final activation of the complex in the nucleus. The nuclear export of CycB1 is required for the cytoplasmic accumulation of the protein at interphase before the onset of mitosis in mammalian cells [28,29]. Although CycB is devoid of a classical nuclear localization signal (NLS), it is intrinsically imported into nucleus in a less efficient manner at interphase during the premeiotic stage (G_2_ stage) [50]. This is considered to be important to sequester CycB temporally out of the cytoplasm to avoid precocious activation of the complex in the case of DNA damage [27,51]. After passing through the DNA damage checkpoint, the CycB-CDK1 complex is exported in the cytoplasm. While the CycB is staying in the cytoplasm, the complex formation with CDK1 is further progressing. In addition, the CycB is phosphorylated at the multiple sites by Plk1 in the cytoplasm. This inhibits the nuclear export of the protein, and consequently promotes its nuclear import [52]. The Cdc25 can be also activated by the Plk1 in the cytoplasm before acting in the nucleus [53]. Subsequently, the CycB-CDK1 is transported into the nucleus again. Then, the phosphate groups on Thr^14^ and Tyr^15^ of the CDK1 are removed by Cdc25 presence in the nucleus. Consequently, the CycB-CDK1 complex is fully activated at the onset of mitosis and meiosis [24,54,55]. In the premeiotic cells depleted of the Nup62 complex, we showed that the CycB was not exported to the cytoplasm and remained to be localized in the nucleus at the latest stage of spermatocytes prior to the onset of meiosis. Therefore, the previous step of active kinase complex formation was insufficiently executed in the cytoplasm before the onset of meiosis. We believe that this is responsible for the failure of meiotic initiation. Overexpression of CycB partially rescued the cell cycle arrest of the *Nup62*-depleted cells. Increased level of CycB induced in cytoplasm possibly provided a threshold of the CycB-CDK1 or above, which can partially rescue the cell cycle arrest. However, we also admitted that the overexpression of CycB was not able to rescue the cell cycle arrest completely. It is likely that a considerable fraction of the induced CycB was also imported and left in the nucleus, as observed in the endogenous protein. Thus, the overexpression might have provided only a limited effect. Based on these results, we believe that the failure of meiotic initiation in cells lacking the central channel in NPC results from insufficient supply of active CycB-CDK1. 

### 4.2. Regulation of the Initiation of Male Meiosis by the Nuclear-Cytoplasmic Transport of CycB-CDK1 

**Figure 9:**
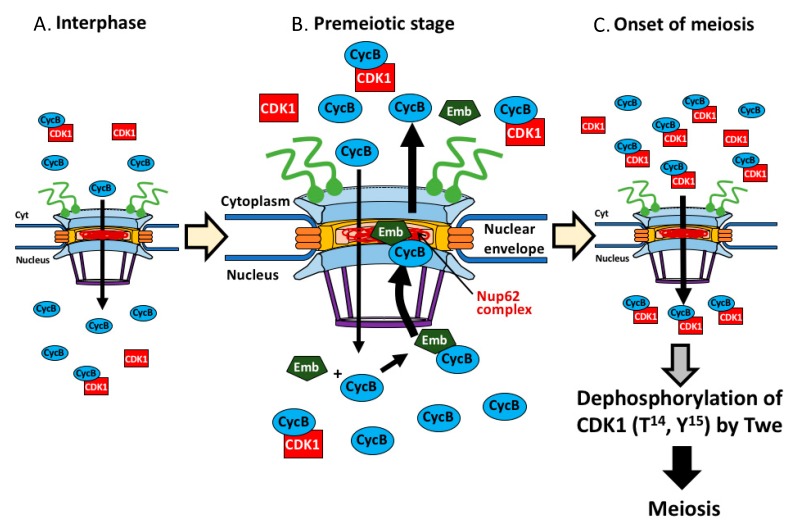
Model showing regulation of initiation of meiosis I by nuclear export of CycB, mediated by the Nup62 complex of NPC and Emb in *Drosophila* male. CycB was temporally entered the nucleus at interphase (growth phase of spermatocyte) (**A**), associates with Emb, and the protein complex is transported back to the cytoplasm via the central channel in the NPC at premeiotic stage before meiosis (**B**). This requires interaction with the Nup62 complex. Owing to this protein export, sufficient amount of the CycB-CDK1 complex is formed in the cytoplasm. Subsequently, the complex is imported to the nucleus again to remove phospho groups of the CDK1 at T^14^ and Y^15^ by Twe (**C**). At the end, the active kinase complex in the nucleus triggers meiosis I in *Drosophila* male.

On the basis of our findings and previous results, we proposed that nuclear export of CycB, mediated by the CRM1/Emb and Nup62 complex in the central channel of NPC, plays a critical role in the initiation of male meiosis in *Drosophila*. The nuclear export of the CycB, which was initially imported at interphase before onset of meiosis enables the formation of a sufficient amount of the CycB-CDK1 complex in the cytoplasm. The formation of a considerable amount of the pre-complex in the cytoplasm is required for the subsequent completion of a sufficient amount of active complex in the nucleus. These are essential steps to trigger meiotic division I (Figure 9). First, exportin (CRM1/Emb) binds to the CycB-CDK1 complex and/or free CycB, which has precociously entered nucleus during the growth phase of spermatocytes corresponding to the G_2_ phase. Second, the exportin-CycB-CDK1 complex moves to the NPC. Third, the complex is selected and transported through the NPC toward the cytoplasm, interacting with the Nup62 complex in the central channel. After passing through the NPC, the exportin is released from the complex in the cytoplasm. After sufficient amount of the CycB-CDK1 complex are formed in the cytoplasm, the complex is imported to the nucleus again. They eventually activated by a removal of the phosphate groups at Thr^14^ and Tyr^15^ of CDK1 by a *Drosophila* Cdc25 orthologue, Twe present in the nucleus [56]. The resultant active CycB-CDK1 complex triggers initiation of meiosis at the end. Previous studies using mammalian cells demonstrated that the nuclear export signal (NES) is present in a domain called the cytoplasmic retention signal (CRS) in CycB. The NES of CycB is conserved from echinoderm until higher eukaryotes including vertebrates, *Drosophila*, but not present in the budding yeast or the fission yeast [57]. One of the exportins, CRM1, recognizes the NES and exports CycB toward the cytoplasm [28,58,59,60]. In this study, we demonstrated that a *Drosophila* CRM1 encoded by *emb* plays an indispensable role in CycB export toward the cytoplasm. Nup62 possesses FG-repeats, which bind to NTF2 required for nuclear-cytoplasmic transport [61]. Binding of the FG-repeats in Nup62 to the CRM1-cargo protein allows the complex to accomplish nuclear-cytoplasmic transport in mammalian cells [62,63]. We believe that a conserved regulatory mechanism involving these factors plays a critical role in meiotic initiation in *Drosophila* males.

### 4.3. Cell Type-Specific Role of the Nup62 Complex, Which Allows the NPC to Fulfill the Selective Transport of the Key Regulator of Cell Cycle 

Although the NPCs had been considered to be static complexes of ubiquitous composition, recent studies have provided evidence that they are more dynamic structures that vary between different cell types [64]. Among the NPC components, the central channel consisted of the Nup62 complex proteins, rather than the NPC scaffold, takes a role in selective transport of specific target proteins between the nucleus and the cytoplasm. The interaction of Nup62 with importin β1 is required for nuclear import of the glucocorticoid receptor-Hsp90 complex [62]. A previous study demonstrated that the Muc1 is imported into the nucleus by a mechanism involving in binding to Nup62 in mammalian cells [65]. Furthermore, another recent study identified the Nup62 as a novel regulator of nuclear import of p63 that is an essential transcription factor for proliferation and maintenance of the undifferentiated status of human carcinoma cells. The authors further demonstrated that the phosphorylation of Nup62 by the Rho-kinase can alter the interaction between the nucleoporin and the p63 [66]. Their finding provided evidence suggesting that the NPC can be utilized to determine cell fate. Our current study also provides another evidence that supports this idea. Four rounds of mitosis of spermatogonia generate a cell unit called the cyst consisting of 16 spermatogonia. The cells differentiate into spermatocytes that launch meiosis later. Depletion of the Nup62 in the spermatogonia did not have any detectable effect on cell proliferation. This result contrasts to the cell cycle arrest of the premeiotic spermatocytes depleted for Nup62. After the differentiation of spermatogonia to spermatocytes, the cell cycle regulation by the nucleo-cytoplasmic transport of CycB via the Nup62 complex was altered. 

### 4.4. Other Possible Roles of Nup62 in Regulation of CycB Expression before Meiosis

In association with failure of CDK1 activation, we observed abnormal distribution of the regulatory subunit CycB before male meiosis. Therefore, we interpreted that this mislocalization was responsible for insufficient cytoplasmic accumulation of the complex and for the loss of its activation at the end. However, we cannot exclude the possibility that CycB expression was also down-regulated in the *Nup62*-depleted cells. *cycB* transcription is dependent on a transcription factor encoded by *aly* in *Drosophila* spermatocytes before meiosis [20,67,68]. Several previous studies have reported that NPC interacts with chromatin regions possessing higher transcriptional activity [69,70,71]. Furthermore, some of the Nups are involved in the regulation of transcription. For example, Nup62, Nup98, and Nup50 are required for the transcription of cell cycle genes [71]. Recently, genetic evidences showed that Nup62 is involved in chromatin organization and transcriptional regulation [72]. Therefore, it would be interesting to understand whether transcription of *cycB* is affected by the depletion of the Nup62 complex. 

The nuclear export of *cycB* mRNA was inhibited in cells lacking central channel components. Mammalian cells possess two types of RNA transport systems: one is dependent on CRM1 and the other is responsible for Nxf1-Nxt1 [73]. The translation of mRNA encoding CycB is further regulated by the RNA binding proteins, Fest and Rbp4, such that the proteins are synthesized immediately prior to male meiosis [74]. Determination of the intracellular localization of the *cycB* mRNA in the *Nup62*-depleted premeiotic spermatocytes and quantitation of the protein level of CycB are warranted in the future.

### 4.5. Concluding Remarks

The major findings in this study are as follows: (1) all the components of the Nup62 complex are specifically required for the initiation of meiotic division; (2) the Nup62 complex is required for nuclear export of CycB; (3) the nuclear export of CycB is mediated by Emb in *Drosophila* spermatocytes.

## Supplementary Materials

The following are available online at https://www.mdpi.com/2073-4409/9/2/270/s1, Figure S1: qRT-PCR experiments to confirm that UAS-RNAi stocks used in this study can deplete the relevant mRNAs efficiently, Figure S2: Immunostaining of premeiotic spermatocytes to confirm that Nup54, another component of Nup62 complex is not localized on nuclear envelope in the Nup62-depleted spermatocytes, Figure S3: Intact cysts consisted of early spermatocytes generated from mitoses of spermatogonia and those of post-meiotic cells derived from spermatocytes depleted of Nup62.
