# Supplementary material for: Nuclear Export of Cyclin B Mediated by the Nup62 Complex Is Required for Meiotic Initiation in Drosophila Males
Source: Cells. 2020 Jan 22;9(2):270. doi: 10.3390/cells9020270 (PMC7072204; doi:10.3390/cells9020270)
Supplement: Supplementary file 1 [file cells-09-00270-s001.pdf]

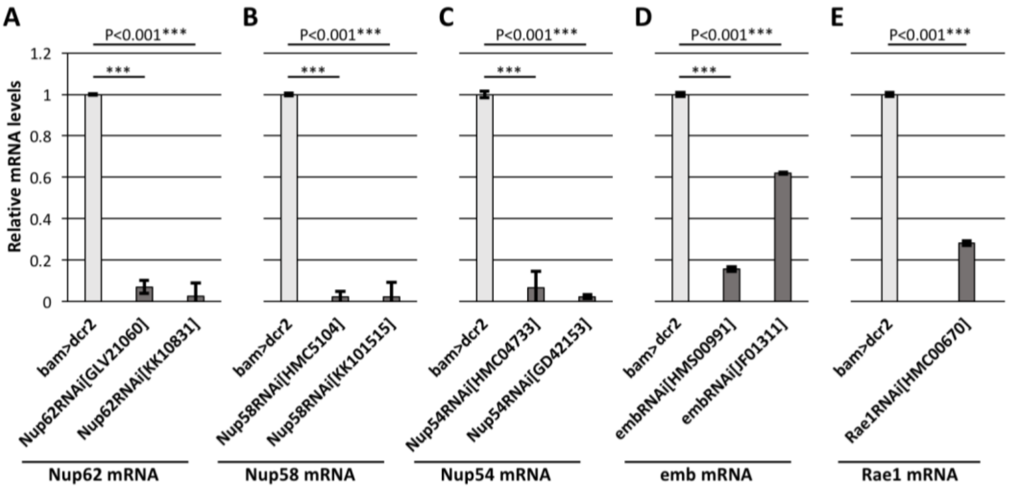

Figure S1: qRT-PCR experiments to confirm that UAS-RNAi stocks used in this study can deplete the relevant mRNAs efficiently.

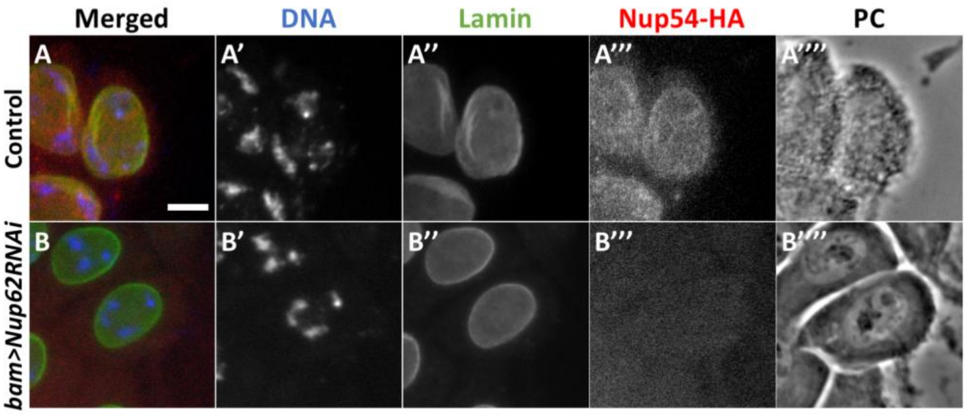

Figure S2: Immunostaining of premeiotic spermatocytes to confirm that Nup54, another component of Nup62 complex is not localized on nuclear envelope in the Nup62-depleted spermatocytes.

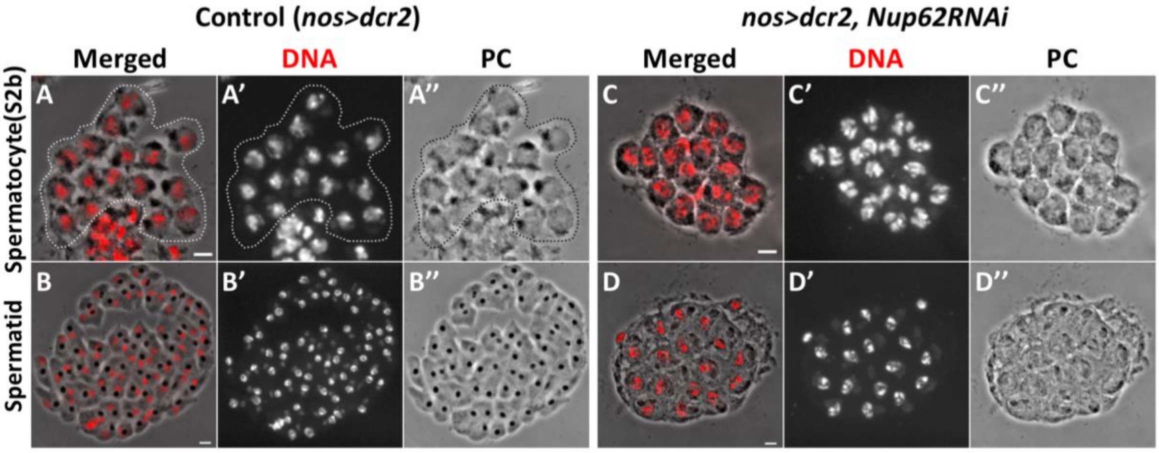

Figure S3: Intact cysts consisted of early spermatocytes generated from mitoses of spermatogonia and those of post-meiotic cells derived from spermatocytes depleted of Nup62.
